# Evaluation of the efficacy of electrospun gelatin/polycaprolactone nanofiber membranes in repairing beagle dog buccal mucosa defects and a comparative study with acellular dermal matrix

**DOI:** 10.3389/fbioe.2026.1800763

**Published:** 2026-05-26

**Authors:** Kun Song, Yunyang Liao, Xu Dong Wang, Gensen Zou, Bin Shi, Yue Huang

**Affiliations:** 1 Department of Oral and Maxillofacial Surgery, The First Affiliated Hospital of Fujian Medical University, Fuzhou, China; 2 Department of Oral and Maxillofacial Surgery, National Regional Medical Center Binhai Campus of the First Affiliated Hospital, Fujian Medical University, Fuzhou, China; 3 Fujian Key Laboratory of Oral Disease, School and Hospital of Stomatology, Fujian Medical University, Fuzhou, China; 4 School and Hospital of Stomatology, Fujian Medical University, Fuzhou, China; 5 Laboratory of Facial Plastic and Reconstruction, Fujian Medical University, Fuzhou, China; 6 Department of Oral and Cranio-Maxillofacial Surgery, Shanghai Ninth People’s Hospital, School of Medicine, Shanghai Jiao Tong University, Shanghai, China

**Keywords:** acellular dermal matrix, buccal mucosal defect, electrospinning, gelatin, nanofibrous membrane, polycaprolactone, tissue engineering

## Abstract

**Objective:**

This study aimed to evaluate the efficacy of electrospun gelatin/polycaprolactone (GT/PCL) nanofibrous membranes in repairing full-thickness buccal mucosal defects in beagle dogs, compared with clinically used acellular dermal matrix (ADM) membranes, thereby providing experimental evidence for clinical translation.

**Methods:**

First, a stable, full-thickness buccal mucosal defect model (2.5 cm × 1.5 cm) was established in beagle dogs using individually designed and fitted palatal protectors. A stepwise bilateral self-paired design was then adopted. In Part I, GT/PCL membranes were implanted into one side of the defect (n = 3), with the contralateral untreated side serving as the control. Macroscopic observations were performed at postoperative days 7 and 14. At day 14, histological assessments—including hematoxylin–eosin (HE) staining, Masson’s trichrome staining, immunohistochemical staining for CD34 and α-smooth muscle actin (α-SMA), along with hydroxyproline (Hyp) quantification—were used to assess wound shrinkage, epithelial regeneration, collagen formation, angiogenesis, and myofibroblast-associated remodeling. In Part II, GT/PCL and ADM membranes were separately implanted into bilateral defects in an additional 9 beagle dogs, which were equally allocated to day 7, day 14, and day 28 endpoint subgroups (n = 3 per time point), followed by comparative analyses of the same outcome measures.

**Results:**

The individually tailored palatal protectors effectively shielded the implanted materials. Compared with the control, GT/PCL membranes significantly accelerated wound healing, evidenced by a lower wound shrinkage rate (P < 0.05), thicker regenerated epithelium (P < 0.05), higher collagen volume fraction and Hyp content (P < 0.05), increased neovascularization (P < 0.05), and reduced α-SMA expression (P < 0.05). In comparison with ADM, GT/PCL membranes promoted significantly greater early epithelial ingrowth and higher collagen-related indices at days 7 and 14 (P < 0.05). However, wound shrinkage rate, neovascularization (CD34-positive vessels), and α-SMA expression did not differ significantly between the two groups (P > 0.05). By day 28, most outcomes were comparable between GT/PCL and ADM.

**Conclusion:**

GT/PCL electrospun nanofibrous membranes exhibit good biocompatibility and effectively support repair of buccal mucosal defects. Compared with spontaneous healing, GT/PCL improved early wound repair. Compared with ADM, GT/PCL promoted earlier epithelial ingrowth and collagen-related matrix deposition during the early phase of healing. However, because most differences between GT/PCL and ADM were attenuated by postoperative day 28, the present findings support an early reparative advantage of GT/PCL rather than definitive long-term superiority over ADM.

## 1 Introduction

The buccal mucosa is essential for mastication, speech, and maintaining a protective barrier. However, its anatomical and functional exposure within the oral cavity also makes it susceptible to pathological conditions such as lichen planus, leukoplakia, ulcers, and squamous cell carcinoma (Jia et al., 2024). Surgical removal of these lesions often creates mucosal defects. Inadequate repair of such defects can lead to postoperative pain, infection, bleeding, and functional limitations including trismus due to scar contracture (Cheng et al., 2025).

Clinically, the reconstruction of buccal mucosal defects has traditionally depended on autologous tissue grafts, such as skin, mucosal, or musculocutaneous flaps. While these methods can mitigate wound contraction, reduce infection risk, and partially restore function, they are associated with notable drawbacks. These include technically demanding and lengthy procedures, donor-site morbidity, and frequent mismatches in the morphology and microstructure between the graft and the recipient site, ultimately compromising functional and aesthetic outcomes (Liu et al., 2021).

Advances in biomaterials and tissue engineering have introduced alternatives like acellular dermal matrix (ADM), collagen membranes, and hydrogels for oral mucosal repair (Wang et al., 2023; Aydin et al., 2025). These innovative materials offer significant advantages by eliminating the need for a secondary donor site and promoting guided tissue regeneration (GTR) (Barzegar et al., 2022). Among them, ADM is a collagen-rich biological scaffold derived from decellularized dermis, which has been clinically used because it can provide structural support for cell infiltration and tissue remodeling while avoiding donor-site morbidity (Shridharani and Tufaro, 2012). However, as a naturally derived material, ADM still has limitations, including restricted tunability of degradation and mechanical properties, potential variability related to tissue source and processing, and incomplete adaptation to the highly dynamic oral environment (Pabst et al., 2022). In addition, the highly dynamic, moist, and microbe-rich environment of the oral cavity presents substantial challenges, making it difficult to precisely control the degradation profile and mechanical performance of implanted materials (Chen et al., 2023; Li et al., 2023). Furthermore, the efficacy, safety, and consistency of currently available materials can vary, limiting their broad application (Zhang et al., 2024). Therefore, an ideal biomaterial for buccal mucosal repair should not only exhibit excellent biocompatibility, but also support early epithelial migration, withstand the moist and mechanically active intraoral environment, and promote orderly extracellular matrix deposition during healing (Zhang et al., 2025).

Gelatin (GT), a derivative of collagen, offers excellent biocompatibility and cell-adhesive properties due to its retained amino acid sequences, alongside low immunogenicity (Wosicka-Frąckowiak et al., 2024). Polycaprolactone (PCL) is a synthetic, biodegradable polymer known for its tunable mechanical strength and degradation rate (Pan and Brassart, 2023). Electrospinning technology facilitates the fabrication of nanofibrous scaffolds that effectively mimic the native extracellular matrix (ECM), providing a high surface-area, porous structure that is ideal for cell attachment, proliferation, and signaling (Muresan et al., 2024). By integrating these advantages, GT/PCL composite nanofiber membranes fabricated via electrospinning have shown promise in balancing mechanical support, degradation kinetics, and bioactivity, demonstrating potential for tendon, bone, and skin repair (Zhang et al., 2022). For buccal mucosal repair, this combination may be particularly advantageous because the GT component can favor epithelial cell and fibroblast adhesion, whereas the PCL component can improve handling properties, structural stability, and persistence of the membrane during the early postoperative period (Schulz et al., 2014). Thus, compared with ADM, GT/PCL may provide a more tunable biomimetic scaffold that better matches the biological and mechanical requirements of oral mucosal healing. A specific “sandwich-structured” GT/PCL membrane, fabricated using an *in-situ* crosslinking technique, has shown particularly encouraging results *in vitro* (Angarano et al., 2013; Guo et al., 2020). Its GT-rich functional layer has been shown to support the adhesion, proliferation, differentiation, and ECM deposition of keratinocytes and fibroblasts, outperforming clinical-grade ADM in some studies and even guiding human oral keratinocytes to form a multi-layered, mucosa-like epithelium (Schulz et al., 2014; Jedrusik et al., 2018; Mahmoud et al., 2023).

Despite these promising *in vitro* findings, the *in vivo* efficacy of this GT/PCL membrane for repairing buccal mucosal defects in a large animal model, and its comparative performance against ADM, remain unverified. This lack of translational evidence represents a major barrier to its clinical adoption. In addition, because the present study was designed as a stepwise preclinical evaluation, we first assessed whether GT/PCL could improve healing relative to spontaneous repair in untreated control defects, and then performed a direct comparison with ADM as a clinically relevant biomaterial comparator. Therefore, this study aims to address this significant gap in knowledge. A buccal mucosal defect model was established in beagle dogs to systematically evaluate, for the first time in a large animal model, the regenerative performance of the GT/PCL nanofiber membrane and to conduct a direct, comparative assessment against ADM. The findings from this investigation are intended to clarify whether GT/PCL provides an early reparative advantage in oral mucosal healing and to provide preclinical evidence for its potential clinical translation.

## 2 Materials and methods

### 2.1 Materials

The GT/PCL nanofibrous membranes were provided by NEO MODULUS (Suzhou China) Medical Technology Co., Ltd. The fabrication method followed the study by (Angarano et al., 2013). utilizing electrospinning and *in situ* cross-linking technology. This process involved bonding two outer layers of gelatin (GT) nanofibers (approximately 400 nm in diameter) to a central polycaprolactone (PCL) layer in a molten state, followed by further processing to form a sandwich-like structure. All test materials were sterilized with ethylene oxide and packaged in double sterile packaging. The acellular dermal matrix (ADM) membrane was purchased from Yantai Zhenghai Biotechnology Co., Ltd. (Yantai, China). It is a collagen-based matrix membrane produced through the decellularization of bovine dermis.

### 2.2 Animals

This study was approved by the Ethics Committee of Fuzhou General Hospital of Nanjing Military Command. All experimental personnel held the Fujian Province Laboratory Animal Practitioner Certificate (No. 2141170). All procedures strictly adhered to the relevant guidelines of the Ministry of Science and Technology’s 2006“Guideline for the Care and Use of Laboratory Animals.” Twelve male beagle dogs, aged 1–1.2 years and weighing 10–12.5 kg, were purchased from Fuzhou Zhenhe Laboratory Animal Technology Development Co., Ltd. (Fuzhou, China) [Laboratory Animal Production License No. SCXK(MIN)2018-0001]. Standard feeding cages were used for housing, and all animals were appropriately marked.

### 2.3 Experimental groups and surgical design

**FIGURE 1:**
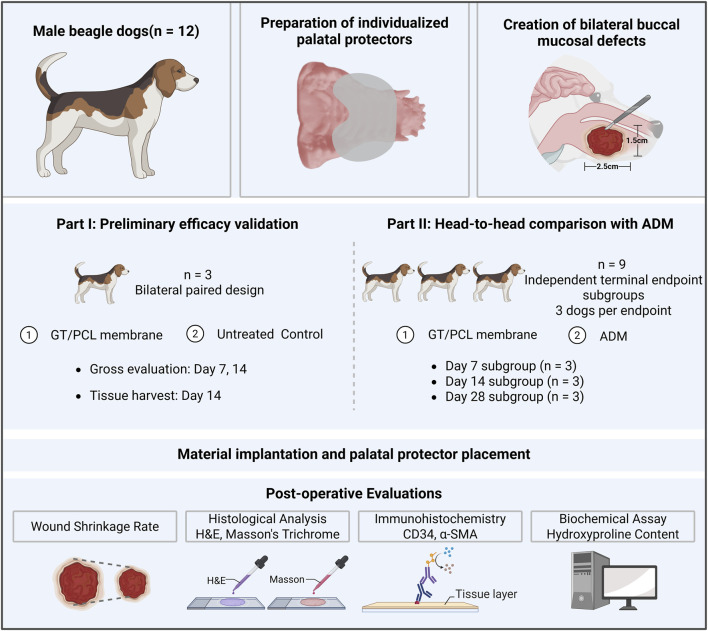
Schematic illustration of the two-part experimental design in the beagle buccal mucosal defect model. A protected bilateral buccal mucosal defect model was established in beagle dogs using individualized palatal protectors. In Part I, bilateral defects were created in 3 beagle dogs, with one side implanted with the GT/PCL membrane and the contralateral side left without material implantation as the control. Gross healing was assessed at postoperative days 7 and 14, and tissue samples were harvested at day 14 for histological, immunohistochemical, and biochemical analyses. In Part II, bilateral defects were created in 9 additional beagle dogs equally allocated to independent terminal endpoint subgroups at postoperative day 7, day 14, and day 28 (n = 3 per time point), with one side implanted with the GT/PCL membrane and the contralateral side implanted with ADM. Gross and tissue-level outcomes were evaluated at the designated endpoint for each subgroup. Outcome measures included gross observation, wound shrinkage rate, HE staining, Masson’s trichrome staining, CD34 immunohistochemistry, α-SMA immunohistochemistry, and hydroxyproline quantification.

The overall study design is illustrated in Figure 1. The study employed a stepwise bilateral self-paired design.

#### 2.3.1 Part I: preliminary efficacy evaluation

A self-paired design was used with three beagle dogs. Bilateral defects in each dog were randomly assigned using a random number table: one side was implanted with the GT/PCL membrane, while the contralateral side received no implanted material and served as the control. Gross healing was assessed at postoperative days 7 and 14. Tissue specimens were harvested at day 14 for histological, immunohistochemical, and biochemical analyses.

#### 2.3.2 Part II: head-to-head comparative study with ADM

To further evaluate its clinical translation potential, nine additional beagle dogs were included. These animals were equally allocated to postoperative day 7, day 14, and day 28 endpoint subgroups (n = 3 per time point). Bilateral full-thickness buccal mucosal defects were created in each dog. Through random assignment, one buccal defect in each dog was implanted with the GT/PCL membrane, and the contralateral defect was implanted with an acellular dermal matrix (ADM) membrane. Gross healing and tissue-level outcomes were evaluated at the designated postoperative endpoint for each subgroup. This terminal time-point design was adopted because tissue harvesting for histological and biochemical analyses required euthanasia at each designated endpoint.

### 2.4 Establishment of the beagle buccal mucosa defect model

#### 2.4.1 Fabrication of individualized palatal guards

One beagle dog with representative jaw morphology was selected. Anesthesia was induced by intramuscular injection of xylazine hydrochloride (0.4 mL/kg). Intraoral scanning was performed to obtain digital data of the dental arch, which was used to print a 3D resin model. Several custom trays for the posterior region were fabricated using light-cured modeling sheets based on this model. Alginate impressions were taken for each experimental dog and poured with super-hard stone. On the resulting casts, a space of approximately 5 mm was reserved on the buccal alveolar mucosa from the mesial of the third premolar to the distal of the fourth premolar bilaterally. Individualized transparent resin palatal guards were then fabricated using an air-pressure molding machine to prevent mechanical damage to the implanted materials caused by physiological activities (Supplementary Figure S1).

#### 2.4.2 Surgical creation of defects and material implantation

A rigid, semi-transparent plastic sheet was trimmed into a 2.5 cm × 1.5 cm rectangle to serve as a static surgical guide, which was sterilized with ethylene oxide. Beagle dogs were anesthetized as described previously. After the loss of pedal and corneal reflexes, the dog was positioned in lateral recumbency on the operating table. The oral cavity and facial skin were disinfected three times with iodophor cotton balls, and a surgical drape was placed. A mouth gag was positioned distal to the canine teeth to achieve adequate mouth opening. A retractor was used to fully expose the buccal mucosa. The surgical guide was placed on the buccal alveolar mucosa approximately 2–3 mm below the region from the third to the fourth premolar. It guided the incision through the mucosal epithelium and lamina propria to the submucosa, creating a full-thickness rectangular defect measuring 2.5 cm × 1.5 cm. Spot electrocautery was used for hemostasis. The repair method for each side was determined using a random number table. The GT/PCL nanofibrous membrane was fully hydrated with sterile saline, trimmed to an appropriate size, and secured with interrupted sutures. The ADM membrane was similarly hydrated, trimmed, and sutured in place. Following the manufacturer’s instructions, an iodoform gauze pack was placed over the ADM and secured with a purse-string suture. Finally, the individualized palatal guard was sutured to the palate to protect the surgical site (Supplementary Figure S2).

### 2.5 Observation and detection indicators

#### 2.5.1 Gross observation and wound shrinkage rate calculation

At specified post-operative time points (Part I: 7, 14 days; Part II: 7, 14, 28 days), the wound site was observed and photographed. ImageJ software was used to calculate wound area and shrinkage rate. The palatal protective plate was removed, and the operative area was exposed and flattened using a mouth gag and surgical retractors. The morphology, color, texture, and surface condition of the newly formed oral mucosa area were observed. High-resolution photographs were taken perpendicular to the wound surface with a digital single-lens reflex camera, using a standard surgical ruler as a reference. ImageJ software was employed to calculate the wound area. The wound shrinkage rate was calculated using Formula 1:

$$\text{Wound shrinkage rate}=\left[\left(\text{Original wound area}-\text{Wound area at observation time point}\right)\;/\text{ Original wound area}\right]\times 100\%$$ (1)

#### 2.5.2 Tissue specimen collection and processing

At specified postoperative time points (Part I: 14 days; Part II: 7, 14, 28 days), tissue specimens encompassing the surgical site and 2–3 mm of adjacent normal tissue were harvested from the margin of the healing area. The incision depth reached 6–8 mm into the submucosa. After rinsing with saline, specimens were divided into two parts using ophthalmic scissors. One part was placed in cryovials and stored at −80 °C for future use. The other part was fixed in 10% neutral formalin solution at room temperature for 24 h.

For subsequent processing, a portion of the tissue was stored at −80 °C in an ultra-low temperature freezer (MDF-U53V, SANYO, Japan) for biochemical analysis. Another portion, fixed in 10% formalin, was used for paraffin embedding and sectioning.

#### 2.5.3 Paraffin embedding and sectioning

Tissues were removed from formalin, rinsed under running water, and trimmed to an appropriate size. They were then sequentially immersed in 50%, 75%, 85%, 95% ethanol I, 95% ethanol II, 100% ethanol I, and 100% ethanol II, each for 1 h, for graded dehydration. After clearing with n-butanol for 1.5 h, the tissues were infiltrated in wax baths I, II, III, and IV, each for 40 min. Embedding was performed using a semi-automatic rotary microtome (FINESSE E+, Thermo Fisher, United States), and 4 µm thick sections were prepared for subsequent experiments.

#### 2.5.4 Hematoxylin and eosin (HE) staining

The prepared sections were baked in an oven for 1.5 h. Sections were deparaffinized and rehydrated by sequential immersion in xylene I, II, III (15 min each), followed by 100% ethanol I, 100% ethanol II, 95% ethanol, 80% ethanol, 70% ethanol, 50% ethanol, and distilled water (5 min each). After rinsing three times with distilled water, sections were stained with hematoxylin (Solarbio, China) for 8 min and rinsed to remove excess stain. Differentiation was performed with 1% acid alcohol for a few seconds, followed by rinsing under running water. Sections were blued in 1% ammonia water for a few seconds, rinsed with tap water, and counterstained with 0.5% eosin solution for 2 min. Excess eosin was removed by rinsing under running water. Sections were dehydrated through a graded ethanol series (70%, 80%, 95%) to differentiate the eosin color, followed by absolute ethanol for 10 s, cleared in xylene for 1 min, and finally mounted with neutral balsam.

Digital images of the sections were obtained using a slide-scanning system. For each specimen, one representative paraffin section encompassing the healing area was selected for quantitative analysis. Epithelial thickness was measured at five predefined locations within the regenerated epithelium, and epithelial ingrowth length was measured along the advancing epithelial front.

#### 2.5.5 Masson’s trichrome staining

Sections were baked, deparaffinized, and rehydrated to water using the method described above. They were then stained with Masson’s composite staining solution (Servicebio, China) for 5 min and rinsed with running water. Next, they were stained with phosphomolybdic acid solution for 5 min, excess liquid was shaken off, and then stained with aniline blue for 5 min followed by a rinse with running water. Differentiation was performed with differentiation solution for 60 s. Routine dehydration through ethanol, clearing in xylene, and mounting with neutral resin after drying were carried out.

#### 2.5.6 Immunohistochemical (IHC) staining

Selected sections were baked, deparaffinized in xylene, and rehydrated through a graded ethanol series as described previously. Sections were immersed in diluted citrate antigen retrieval buffer (1:100) (Maixin, China) for 2 min and subjected to high-pressure retrieval for 3 min. After cooling under running water, sections were rinsed with PBS three times (3 min each). Excess liquid was shaken off, and a hydrophobic pen was used to draw a circle around the tissue. An appropriate amount of endogenous peroxidase blocker was applied, and slides were incubated at 37 °C for 10–15 min. After rinsing with PBS three times (3 min each) and shaking off excess liquid, a non-specific blocking reagent was applied and incubated at 37 °C for 10–15 min. Excess liquid was shaken off, and primary antibodies (CD34 and α-SMA diluted 1:100; Servicebio, China) were applied. For the negative control, PBS was applied instead. Slides were incubated at 4 °C for 12 h. They were then warmed to room temperature in a 37 °C incubator for 30 min. Secondary antibody was applied and incubated at 37 °C for 10 min. After rinsing with PBS three times (3 min each), tertiary antibody was applied and incubated at 37 °C for 10 min. DAB chromogen solution was applied under microscopic observation, and the reaction was stopped at an appropriate time. Counterstaining was performed with hematoxylin for 30 s, followed by routine dehydration, clearing, drying, and mounting.

For image-based quantification, five randomly selected, non-overlapping microscopic fields within the healing area were analyzed on Masson’s trichrome-, CD34^−^, and α-SMA-stained sections using ImageJ software. For each parameter, measurements were averaged across the five fields or predefined locations to generate one value per animal, and the animal was considered the statistical unit. Image-based quantification was performed in a blinded manner with group allocation concealed from the evaluator.

#### 2.5.7 Enzyme-linked immunosorbent assay (ELISA)

Tissue specimens previously stored at −80 °C were thawed at 4 °C. The surrounding normal tissue was trimmed off, and the sample was rinsed with saline, blotted dry with lint-free paper, and weighed. A volume of PBS solution nine times the tissue weight was added. The tissue was thoroughly homogenized in a tissue grinder. The homogenate was centrifuged at 3,000 rpm for 15 min, and the supernatant was collected for later use.

#### 2.5.8 BCA total protein concentration assay

Protein standards were prepared according to the manufacturer’s instructions. For the assay, 10 μL of each sample was added to a 96-well plate, followed by 40 μL of PBS. After adding the working reagent from the BCA Protein Assay Kit (Beyotime, China), the plate was incubated at 37 °C in the dark for 30 min. Absorbance was measured at 562 nm using a microplate reader (BIO-RAD iMark, Bio-Rad, United States). A standard curve was plotted using the standard concentrations and their corresponding OD values. The total protein content of each sample was calculated based on this standard curve.

#### 2.5.9 Hydroxyproline (Hyp) content analysis

Reagents from the assay kit were allowed to equilibrate to room temperature for 50 min. Standards and samples (diluted 5-fold) were sequentially added to the microplate wells. After adding the enzyme conjugate reagent (Jianglai, China), the plate was incubated at 37 °C in the dark for 1 h. Following sequential washing, color development, and stop solution steps, absorbance was measured at 450 nm. A standard curve was plotted using the standard concentrations and their corresponding OD values. The Hyp content of each sample was calculated based on this standard curve and the total protein content.

### 2.6 Randomization and blinding

For both Part I and Part II, the treatment assigned to each side of the bilateral buccal defect was determined using a random number table. In Part I, the randomized allocation was GT/PCL versus control; in Part II, it was GT/PCL versus ADM. During histomorphometric and immunohistochemical quantification, image-based measurements were performed in a blinded manner with group allocation concealed from the evaluator.

### 2.7 Statistical analysis

Data are presented as mean ± standard deviation (SD). Because both Part I and Part II used a bilateral paired design, comparisons between GT/PCL-treated wounds and the contralateral comparator wounds were performed using paired t-tests. In Part II, the 9 beagle dogs were allocated to postoperative day 7, day 14, and day 28 independent terminal endpoint subgroups (n = 3 per time point). Because animals assigned to different postoperative endpoints constituted independent subgroups rather than repeated longitudinal measurements in the same animals, comparisons between GT/PCL and ADM were performed separately at each time point rather than using a repeated-measures model.

No formal *a priori* power calculation was performed because this study was designed as an exploratory preclinical large-animal study and reliable effect-size estimates for this specific beagle buccal mucosal defect model were not available at the design stage. The sample size was determined based on the exploratory nature of the study, the bilateral paired design used to reduce inter-animal variability, ethical considerations regarding the use of large animals, and feasibility constraints. Because the analyses were exploratory paired comparisons performed separately at each postoperative endpoint, no formal multiple-comparison adjustment was applied, and the findings should therefore be interpreted with appropriate caution. A value of P < 0.05 was considered statistically significant. Statistical analyses were performed using GraphPad Prism 8.0.1 software.

## 3 Results

### 3.1 Stable establishment of the protected beagle buccal mucosal defect model

The individualized palatal protectors were well retained in all beagle dogs throughout the observation period, and all implanted materials remained adequately protected during the early healing stage. No accidental material loss, obvious wound infection, or severe postoperative complications were observed. All animals maintained stable vital signs and tolerated the procedures well, indicating that the protected bilateral buccal mucosal defect model was successfully established and was suitable for subsequent evaluation of biomaterial-assisted healing.

### 3.2 Part I: GT/PCL improved early healing compared with the control defects

**FIGURE 2:**
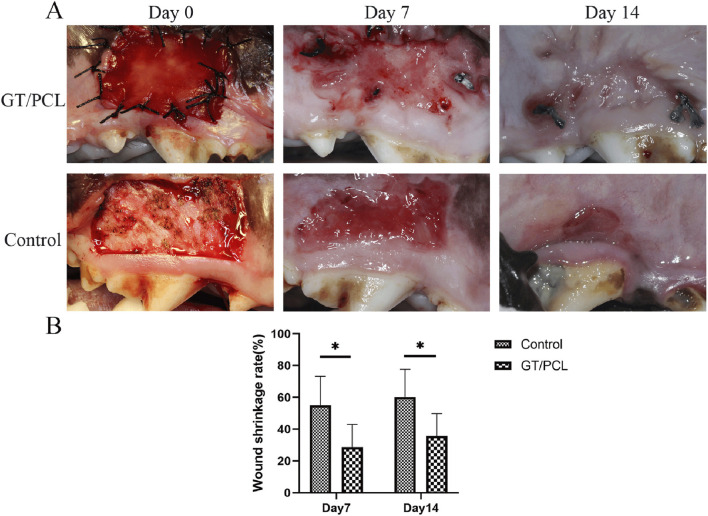
GT/PCL improved gross healing compared with the control defects in Part I. **(A)** Representative gross images of the buccal mucosal defects in the control and GT/PCL groups at postoperative days 7 and 14 in Part I. In Part I, the control defects were contralateral wounds that received no implanted material. **(B)** Quantitative comparison of wound shrinkage rates between the control and GT/PCL groups at postoperative days 7 and 14. Data are presented as mean ± SD. *P < 0.05.

Gross observation showed that wounds treated with the GT/PCL membrane exhibited more favorable early healing than the contralateral control defects. At postoperative day 7, the GT/PCL-treated wounds appeared fuller in contour and more pinkish in color, with more evident epithelial coverage, whereas the control wounds appeared darker red, less full, and showed less obvious surface epithelialization. By postoperative day 14, the GT/PCL-treated wounds had largely healed, apart from mild localized redness around some suture sites, while the control wounds still showed incompletely healed areas. Quantitative analysis further demonstrated that the wound shrinkage rate differed significantly between the two groups at both day 7 and day 14, indicating improved early macroscopic healing in the GT/PCL group (Figure 2).

**FIGURE 3:**
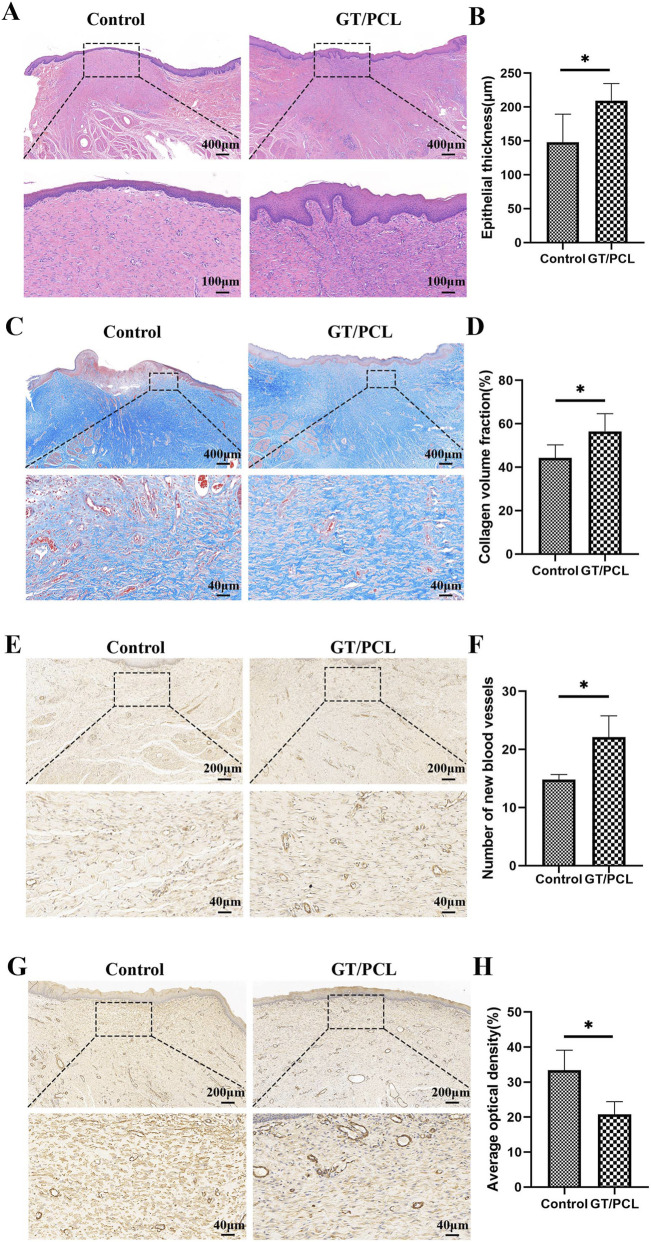
Histological and immunohistochemical validation of improved early healing by GT/PCL at day 14 in Part I. **(A)** Representative hematoxylin and eosin (HE) staining images of healing tissues in the control and GT/PCL groups at postoperative day 14 in Part I. **(B)** Quantification of epithelial thickness. **(C)** Representative Masson’s trichrome staining images. **(D)** Quantification of collagen volume fraction. **(E)** Representative CD34 immunohistochemical staining images. **(F)** Quantification of the number of new vessels. **(G)** Representative α-SMA immunohistochemical staining images. **(H)** Quantification of α-SMA expression as average optical density. In Part I, the control defects were contralateral wounds that received no implanted material. Data are presented as mean ± SD. *P < 0.05.

Histological and immunohistochemical analyses at postoperative day 14 were consistent with the gross findings. HE staining showed that the GT/PCL-treated wounds had a more compact tissue architecture, a thicker regenerated epithelium, and more evident epithelial peg formation. In contrast, the control wounds displayed a much thinner epithelial layer and less organized epithelial regeneration. Quantitative measurement confirmed that epithelial thickness was significantly greater in the GT/PCL group than in the control group (Figures 3A,B). Masson’s trichrome staining showed more abundant collagen deposition in the GT/PCL-treated wounds, with thicker and more regularly arranged collagen bundles than those observed in the control defects (Figure 3C). Quantitative analysis confirmed a significantly higher collagen volume fraction in the GT/PCL group (Figure 3D). Biochemical analysis further showed that hydroxyproline concentration was significantly higher in GT/PCL-treated wounds than in control wounds at postoperative day 14, consistent with the histological findings and supporting enhanced early matrix deposition during healing (Supplementary Figure S3). Compared with unwounded normal buccal mucosa, however, the collagen architecture of the healing tissue remained distinct from that of intact tissue at this stage (Supplementary Figure S4). CD34 immunohistochemistry showed more newly formed vessels in the GT/PCL group than in the control group, and the number of new vessels was significantly increased in GT/PCL-treated wounds (Figures 3E,F). In contrast, α-SMA immunohistochemistry showed lower expression in the GT/PCL group than in the control group, with quantitative analysis confirming significantly reduced average optical density of α-SMA in GT/PCL-treated wounds (Figures 3G,H). Overall, compared with the control defects, GT/PCL-treated wounds showed greater epithelial thickness, higher collagen volume fraction and hydroxyproline content, more newly formed vessels, and lower α-SMA expression at postoperative day 14.

### 3.3 Part II: GT/PCL and ADM showed comparable gross wound shrinkage over time

**FIGURE 4:**
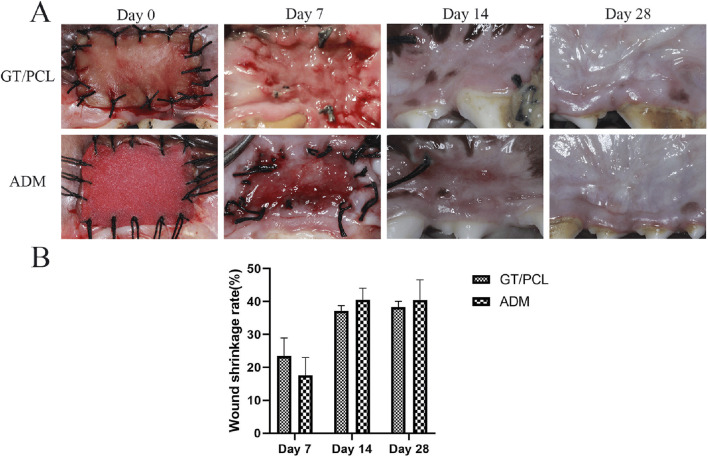
Gross comparison of GT/PCL- and ADM-treated wounds during healing in Part II. **(A)** Representative gross images of buccal mucosal defects treated with GT/PCL or ADM at postoperative days 0, 7, 14, and 28 in Part II. **(B)** Quantitative comparison of wound shrinkage rates between the GT/PCL and ADM groups at postoperative days 7, 14, and 28. Data are presented as mean ± SD. Differences were not statistically significant.

In the head-to-head comparison with ADM, both materials supported progressive wound healing over time. At postoperative days 7 and 14, GT/PCL-treated wounds generally showed a fuller surface appearance and more apparent epithelial coverage than ADM-treated wounds. By day 28, wounds in both groups were macroscopically healed, and the regenerated mucosa in both groups showed color and texture similar to the surrounding tissue. Despite these qualitative differences during the early healing stage, quantitative analysis showed no statistically significant difference in wound shrinkage rate between the GT/PCL and ADM groups at postoperative days 7, 14, or 28 (Figure 4).

### 3.4 GT/PCL promoted earlier epithelial ingrowth than ADM during early healing

**FIGURE 5:**
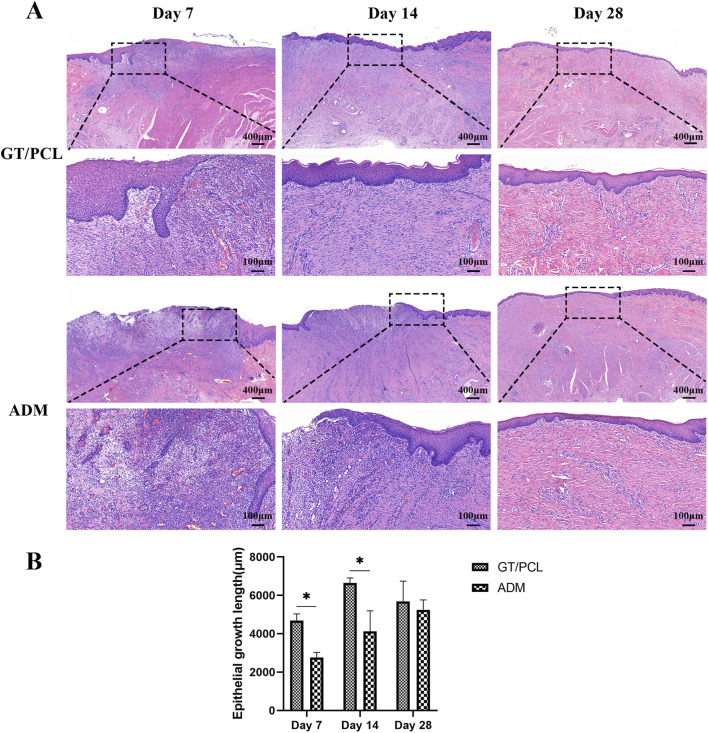
GT/PCL promoted earlier epithelial ingrowth than ADM during healing in Part II. **(A)** Representative HE staining images of healing tissues in the GT/PCL and ADM groups at postoperative days 7, 14, and 28 in Part II. **(B)** Quantification of epithelial ingrowth length in the GT/PCL and ADM groups at postoperative days 7, 14, and 28. Data are presented as mean ± SD. *P < 0.05.

HE staining demonstrated distinct temporal differences in epithelial regeneration between the two treatment groups. At postoperative day 7, GT/PCL-treated wounds already exhibited evident epithelial ingrowth, with a thin but recognizable epithelial layer and basal layer formation. In contrast, ADM-treated wounds showed loose granulation tissue with inflammatory cell infiltration and little to no obvious epithelial ingrowth. At day 14, the GT/PCL-treated wounds were essentially epithelialized, with a compact epithelialized tissue structure and visible epithelial pegs, whereas ADM-treated wounds still showed partially epithelialized areas, with some central regions lacking complete epithelial coverage. By day 28, both groups exhibited continuous epithelial coverage, although histological remodeling was still ongoing when compared with unwounded normal buccal mucosa (Supplementary Figure S4). Quantitative analysis confirmed that epithelial ingrowth length was significantly greater in the GT/PCL group than in the ADM group at postoperative days 7 and 14, whereas this difference was no longer apparent at day 28 (Figure 5).

### 3.5 GT/PCL enhanced early collagen deposition compared with ADM

**FIGURE 6:**
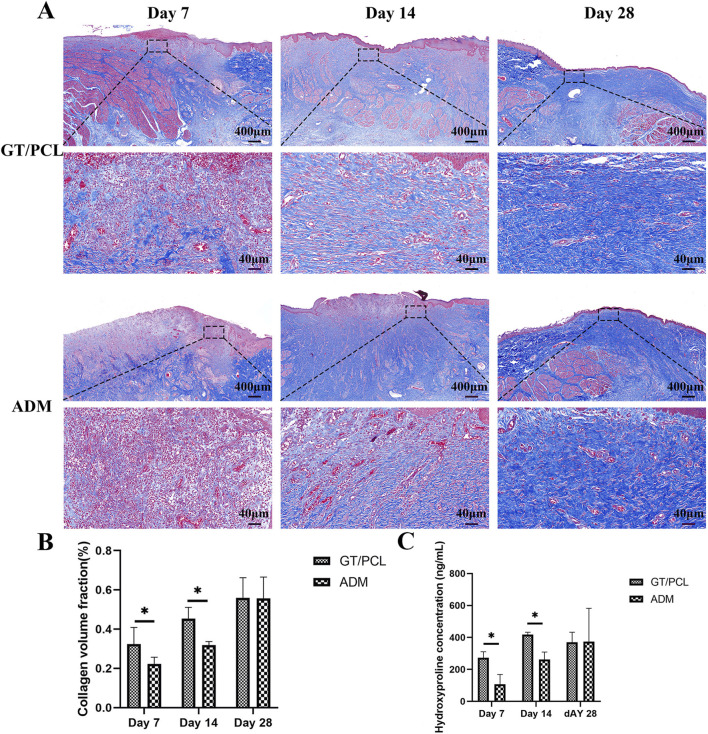
GT/PCL enhanced early collagen deposition compared with ADM in Part II. **(A)** Representative Masson’s trichrome staining images of healing tissues in the GT/PCL and ADM groups at postoperative days 7, 14, and 28 in Part II. **(B)** Quantification of collagen volume fraction in the GT/PCL and ADM groups at postoperative days 7, 14, and 28. **(C)** Quantification of hydroxyproline concentration in the GT/PCL and ADM groups at postoperative days 7, 14, and 28. Data are presented as mean ± SD. *P < 0.05.

Masson’s trichrome staining showed earlier and more pronounced collagen deposition in the GT/PCL-treated wounds than in the ADM-treated wounds. At postoperative day 7, collagen fiber formation was already discernible in the GT/PCL group, whereas collagen deposition in the ADM group remained sparse and less organized. At day 14, the GT/PCL group exhibited thicker and more regularly arranged collagen bundles, whereas collagen fibers in the ADM group were thinner and more disorganized. By day 28, both groups displayed obvious collagen deposition, although collagen bundles in the GT/PCL group appeared more regularly aligned, while the ADM group still showed a relatively less ordered pattern. Quantitative analysis confirmed that the collagen volume fraction was significantly higher in the GT/PCL group than in the ADM group at postoperative days 7 and 14, with no significant difference at day 28. Consistently, hydroxyproline concentration was also significantly higher in the GT/PCL group at days 7 and 14 but became comparable between the two groups at day 28 (Figure 6). These findings were consistent with earlier matrix deposition in the GT/PCL group during healing, whereas the differences between the two groups were no longer evident by day 28. Compared with unwounded normal buccal mucosa, however, the collagen architecture of the healing tissue remained distinct from that of intact tissue at this stage (Supplementary Figure S4).

### 3.6 GT/PCL and ADM showed comparable angiogenesis and α-SMA expression

**FIGURE 7:**
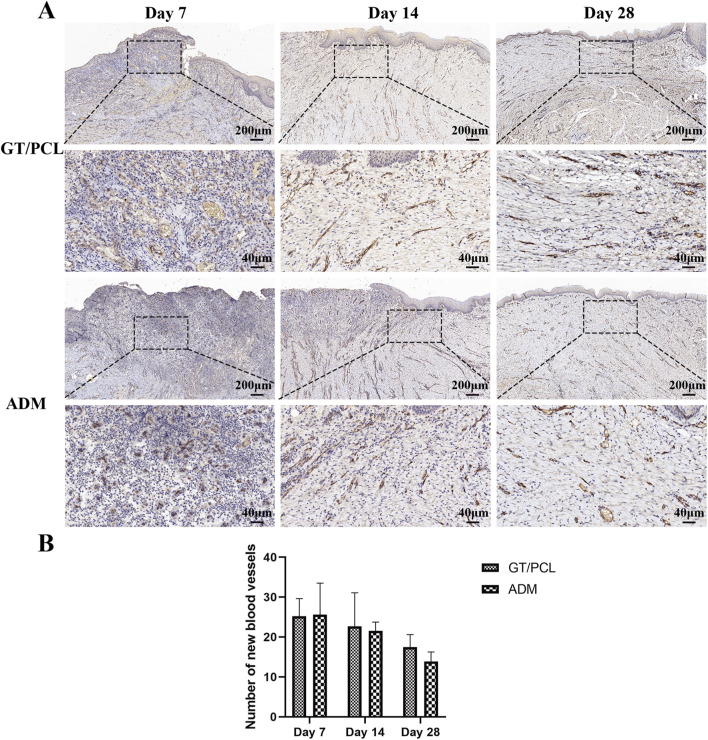
CD34 immunohistochemical staining of GT/PCL- and ADM-treated wounds in Part II. **(A)** Representative CD34 immunohistochemical staining images of healing tissues in the GT/PCL and ADM groups at postoperative days 7, 14, and 28 in Part II. **(B)** Quantification of the number of new vessels in the GT/PCL and ADM groups at postoperative days 7, 14, and 28. Data are presented as mean ± SD. Differences were not statistically significant.

CD34 immunohistochemistry showed abundant neovessel formation in both the GT/PCL and ADM groups during healing. At postoperative days 7 and 14, numerous newly formed vessels were distributed throughout the granulation tissue in both groups, and quantitative analysis showed no statistically significant difference in the number of new vessels between GT/PCL- and ADM-treated wounds at any observation time point (Figure 7).

**FIGURE 8:**
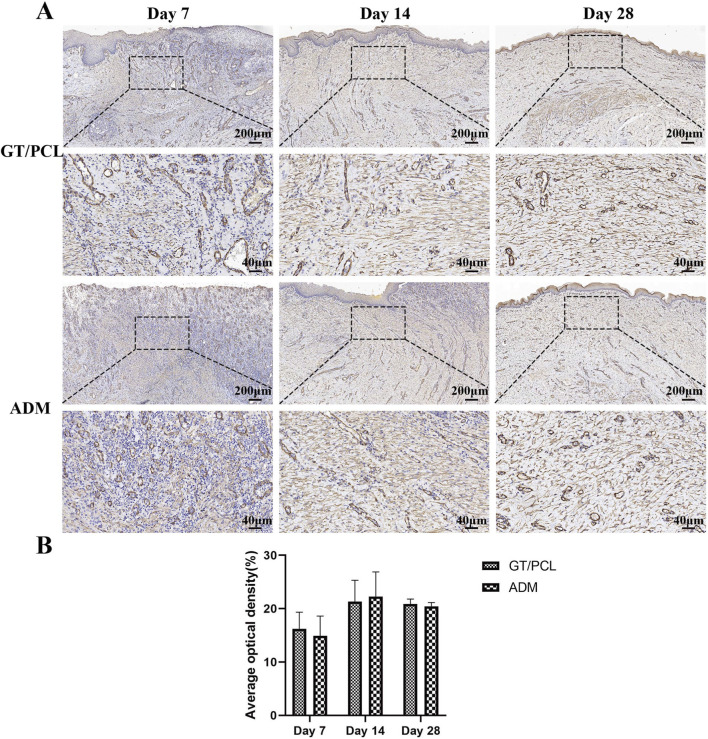
α-SMA immunohistochemical staining of GT/PCL- and ADM-treated wounds in Part II. **(A)** Representative α-SMA immunohistochemical staining images of healing tissues in the GT/PCL and ADM groups at postoperative days 7, 14, and 28 in Part II. **(B)** Quantification of α-SMA expression as average optical density in the GT/PCL and ADM groups at postoperative days 7, 14, and 28. Data are presented as mean ± SD. Differences were not statistically significant.

Similarly, α-SMA immunohistochemistry showed positive spindle-shaped staining areas in both groups during the healing process. At day 7, α-SMA-positive areas were observed mainly near the wound margins in both groups. At day 14, α-SMA expression remained detectable in the epithelialized regions of both groups, while by day 28 evenly arranged spindle-shaped positive areas were present in both healing tissues. Quantitative analysis showed no statistically significant difference in average optical density of α-SMA between the GT/PCL and ADM groups at postoperative days 7, 14, or 28 (Figure 8).

## 4 Discussion

Establishing a stable and reproducible animal model is a crucial prerequisite for evaluating the *in vivo* performance of biomaterials, particularly for intricate anatomical sites like the buccal mucosa, which is susceptible to interference from tongue movement and mastication (Ge et al., 2012; Zhang et al., 2021). This study addressed this challenge by implementing a protective strategy that integrated a customized modified palatal stent with an Elizabethan collar, effectively mitigating early mechanical damage to the implanted material from instinctive animal behaviors. This approach reduced uncontrolled mechanical disturbance during the early healing stage and provided a practical platform for evaluating biomaterial-assisted repair in the oral environment. The successful maintenance of the model in the present study suggests that it may be useful for future preclinical investigations of oral lining mucosal repair.

An ideal material for mucosal repair should fulfill multiple criteria, including good biocompatibility, regenerative support, controllable degradation, and appropriate mechanical properties (Ding et al., 2024; Xu et al., 2025). The gelatin/polycaprolactone (GT/PCL) nanofiber membrane used in this study features a unique “sandwich” structure, which combines the benefits of both GT and PCL (Angarano et al., 2013). In a hydrated state, this material exhibits good flexibility, facilitating ease of trimming and suturing during surgical procedures, thus meeting fundamental operational requirements. Because gelatin is highly hydrophilic and bioactive, the GT-rich functional layer may also favor the absorption of wound exudate and early cell–material interactions at the wound surface, thereby contributing to a microenvironment favorable for repair (Fu et al., 2014; Al-Baadani et al., 2023). In addition, histological observation showed only minimal lymphocyte infiltration after GT/PCL implantation, without evidence of immune rejection or inflammatory fibrous capsule formation, supporting the *in vivo* biocompatibility of the material. Our Part I results showed that GT/PCL-treated wounds exhibited more favorable gross healing, greater epithelial thickness, higher collagen-related indices, more newly formed vessels, and lower α-SMA expression than control defects, indicating that GT/PCL treatment was associated with improved early repair compared with spontaneous healing alone. This interpretation is also supported by previous studies showing that GT/PCL-based electrospun scaffolds can support oral soft-tissue-related cell adhesion, proliferation, and matrix-associated responses *in vitro* (Schulz et al., 2014; Tian et al., 2023).

In the direct comparison with ADM, however, the advantage of GT/PCL was not global across all evaluated parameters. The most consistent difference between the two materials was observed during the early healing phase. GT/PCL-treated wounds showed greater epithelial ingrowth at postoperative days 7 and 14, whereas this difference was no longer apparent at day 28. Therefore, the present findings are better interpreted as evidence of earlier re-epithelialization rather than definitive proof of superior long-term regeneration. By day 28, both groups exhibited continuous epithelial coverage, and the overall macroscopic appearance of the wounds had become broadly comparable. This interpretation is also consistent with the known clinical role of ADM as a biologically active collagen-rich scaffold that can support oral soft-tissue reconstruction, but does not necessarily guarantee superiority over other biomaterials across all healing endpoints (Shi et al., 2012; Tognetti et al., 2021).

Similarly, Masson’s trichrome staining and hydroxyproline quantification showed higher collagen-related indices in the GT/PCL group at postoperative days 7 and 14, whereas the differences between the two groups were no longer evident at day 28. These findings were consistent with earlier matrix deposition in the GT/PCL group during healing. However, increased collagen deposition alone should not be interpreted as unequivocal evidence of improved regeneration, because excessive extracellular matrix and collagen accumulation may also be associated with fibrotic or scar-mediated repair (Coentro et al., 2019; Moss et al., 2022; Mayorca-Guiliani et al., 2025). In the present study, wound shrinkage rate and α-SMA expression were not significantly increased in the GT/PCL group relative to ADM, which argues against a simple interpretation of enhanced scar contraction. Nevertheless, comparison with unwounded normal buccal mucosa indicated that the collagen architecture of the healing tissue had not yet fully recapitulated that of intact mucosa at this stage. Accordingly, the collagen-related findings in the present study are more appropriately interpreted as evidence of accelerated early matrix formation and remodeling rather than definitive restoration of normal tissue structure.

By contrast, no statistically significant differences were found between GT/PCL and ADM in wound shrinkage rate, CD34-positive vessel formation, or α-SMA expression in Part II. Taken together, these findings suggest that the relative advantage of GT/PCL over ADM in the present model was selective rather than global, and was mainly reflected in earlier epithelial ingrowth and matrix deposition rather than in overall wound contraction, angiogenesis, or α-SMA-associated remodeling. This interpretation is consistent with previous reports emphasizing the importance of cell–scaffold interactions and matrix-supportive scaffold design in oral soft-tissue engineering, as well as with direct GT/PCL studies showing favorable responses in oral-related soft-tissue contexts (Coentro et al., 2019; Tian et al., 2023; Wang et al., 2023).

Several limitations of the present study should be acknowledged. First, this was a preclinical exploratory study with a relatively limited number of animals, and Part II was conducted using independent terminal endpoint subgroups rather than repeated longitudinal measurements in the same animals. This design was necessary because tissue harvesting for histological and biochemical analyses required euthanasia at each designated endpoint, but it also limited continuous temporal assessment within the same individual. Second, the absence of gelatin-only or PCL-only comparator groups limited mechanistic interpretation of the individual contribution of each scaffold component. Finally, inflammatory or immune-response analyses were not included, which further limited mechanistic interpretation of the observed early advantage of GT/PCL.

Taken together, the present study indicates that GT/PCL is a biocompatible and promising membrane for buccal mucosal repair. Its principal benefit relative to ADM appears to lie in promoting earlier epithelial coverage and earlier matrix deposition during the early phase of healing. However, because most differences between GT/PCL and ADM were attenuated by postoperative day 28, the present findings support an early reparative advantage of GT/PCL rather than definitive long-term superiority over ADM. Whether these early differences translate into superior long-term tissue quality will require further investigation with longer follow-up and more detailed mechanistic analyses.

## 5 Conclusion

GT/PCL electrospun nanofibrous membranes showed good biocompatibility and effectively supported repair of buccal mucosal defects in a beagle dog model. Compared with spontaneous healing, GT/PCL improved early wound repair in Part I. Compared with ADM, GT/PCL promoted earlier epithelial ingrowth and collagen-related matrix deposition during the early phase of healing in Part II. However, because most differences between GT/PCL and ADM were attenuated by postoperative day 28, the present findings support an early reparative advantage of GT/PCL rather than definitive long-term superiority over ADM. GT/PCL may therefore represent a promising candidate biomaterial for oral mucosal reconstruction, but further studies with longer follow-up and more detailed mechanistic analyses are still required.

## Data Availability

The datasets presented in this article are available from the corresponding authors on reasonable request. Requests to access the datasets should be directed to ayue784@126.com.
