# Supplementary material for: Evaluation of the efficacy of electrospun gelatin/polycaprolactone nanofiber membranes in repairing beagle dog buccal mucosa defects and a comparative study with acellular dermal matrix
Source: Front Bioeng Biotechnol. 2026 May 26;14:1800763. doi: 10.3389/fbioe.2026.1800763 (PMC13245998; doi:10.3389/fbioe.2026.1800763)
Supplement: Supplementary file 1 [file Supplementaryfile1.doc]

**Supplementary Figures**


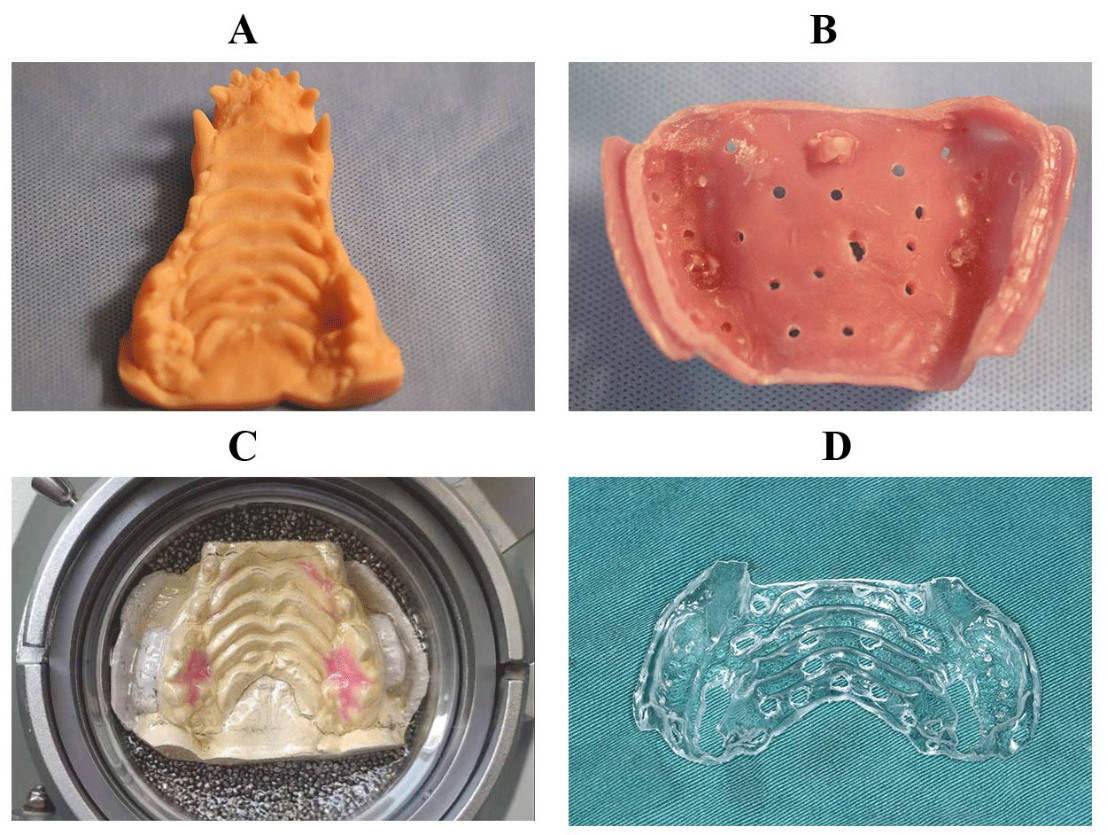


**Supplementary Figure S1. Preparation of the modified individualized palatal protector for beagle dogs.**

(A) Three-dimensional resin model of the beagle maxilla.

(B) Customized tray for the maxillary posterior region.

(C) Fabrication process of the palatal protector using the maxillary posterior model and an air-pressure molding machine.

(D) Final modified individualized palatal protector used to shield the surgical site from mechanical disturbance during healing.


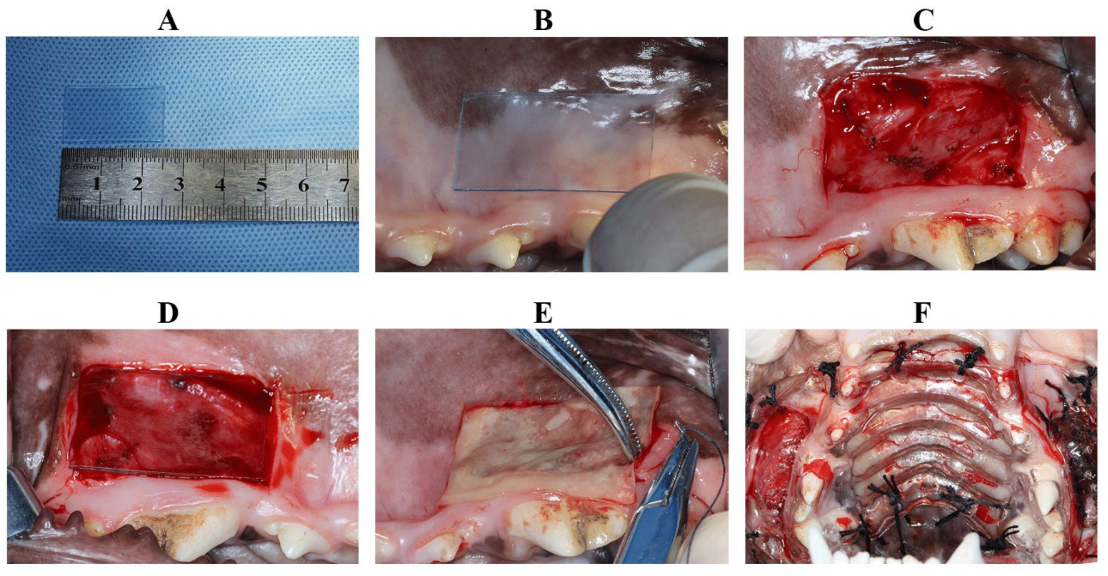


**Supplementary Figure S2. Establishment of the beagle buccal mucosal defect model and material implantation procedure.**

(A) Surgical guide plate used to standardize the defect size.

(B) Buccal mucosal resection guided by the surgical template.

(C) Hemostasis after defect creation.

(D) Standardized full-thickness buccal mucosal defect.

(E) Material implantation into the defect site.

(F) Placement and fixation of the individualized palatal protector after surgery.


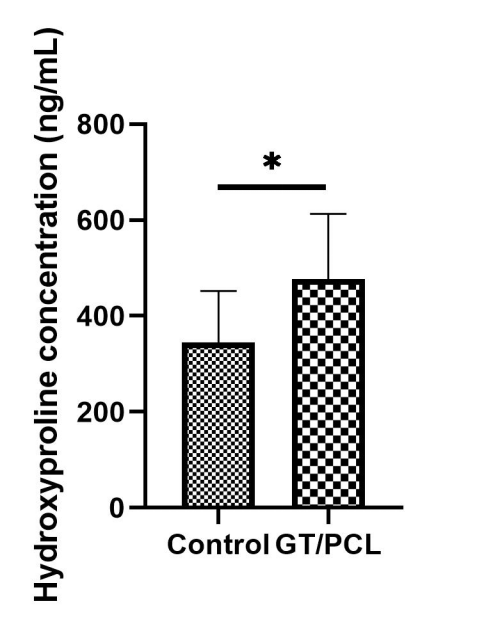


**Supplementary Figure S3. Hydroxyproline content of healing tissues in Part I.**

Quantitative comparison of hydroxyproline concentration between the control and GT/PCL groups at postoperative day 14 in Part I. In Part I, the control defects were contralateral wounds that received no implanted material. Data are presented as mean ± SD. *P < 0.05.


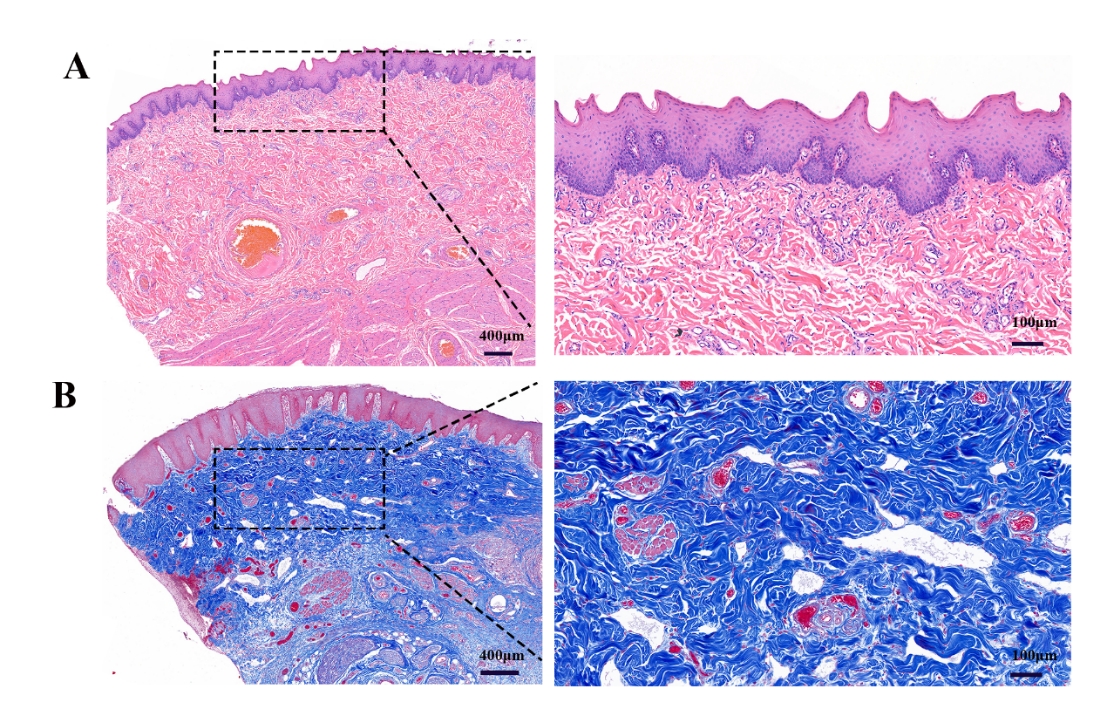


**Supplementary Figure S4. Histological reference of unwounded normal buccal mucosa in beagle dogs.**

(A) Representative HE staining images of unwounded normal buccal mucosa.

(B) Representative Masson’s trichrome staining images of unwounded normal buccal mucosa.

These images are provided as histological references for epithelial morphology and collagen organization when interpreting the healing tissues in Part I and Part II.
